# Discovering Hereditary Risk Through Surveillance: A Prospective Genetic Analysis of Individuals With Familial Pancreatic Cancer

**DOI:** 10.1002/ueg2.70187

**Published:** 2026-02-15

**Authors:** Salvatore Paiella, Erica Secchettin, Livia Archibugi, Raffaele De Luca, Cristiana Bonifacio, Luigi Laghi, Gabriella Lionetto, Anna Caterina Milanetto, Giuliana Sereni, Chiara Coluccio, Gaetano Lauri, Arianna Dal Buono, Margherita Patruno, Giulia Gabriel, Romano Sassatelli, Cecilia Binda, Deborah Bonvissuto, Vera Uliana, Giuseppe Malleo, Giulia Martina Cavestro, Maria Terrin, Stefania Martino, Claudio Pasquali, Matteo De Pastena, Francesco De Cobelli, Valeria Poletti, Elisa Venturini, Marta Puzzono, Alessandro Zerbi, Paolo Giorgio Arcidiacono, Roberto Salvia, Massimo Falconi, Gabriele Capurso, Silvia Carrara

**Affiliations:** ^1^ University of Verona Verona Italy; ^2^ Pancreatic Surgery Unit, Department of Surgery, Dentistry, Paediatrics and Gynecology University Hospital of Verona Verona Italy; ^3^ Department of Surgery, Dentistry, Paediatrics and Gynecology University of Verona Verona Italy; ^4^ Pancreato‐Biliary Endoscopy and Endosonography Division Pancreas Translational and Clinical Research Center IRCCS San Raffaele Scientific Institute Milan Italy; ^5^ Department of Surgical Oncology IRCCS Istituto Tumori “Giovanni Paolo II” Bari Italy; ^6^ Department of Radiology IRCCS Humanitas Research Hospital Milan Italy; ^7^ Dept. of Medicine and Surgery University of Parma Parma Italy; ^8^ Department of Surgery, Oncology and Gastroenterology University of Padua Padua Italy; ^9^ UniCamillus International Medical University in Rome Roma Italy; ^10^ Gastroenterology and Digestive Endoscopy Unit Azienda USL ‐ IRCCS di Reggio Emilia Reggio Emilia Italy; ^11^ Gastroenterology and Digestive Endoscopy Unit Forlì‐Cesena Hospitals Forlì‐Cesena Italy; ^12^ Department of Biomedical Sciences Humanitas University Milan Italy; ^13^ Department of Gastroenterology IRCCS Humanitas Research Hospital Milan Italy; ^14^ Center for Study of Heredo‐Familial Tumors IRCCS Istituto Tumori “Giovanni Paolo II” Bari Italy; ^15^ Genomica s.r.l., Department of Cancer Genetics Rome Italy; ^16^ Medical Genetics University Hospital of Parma Parma Italy; ^17^ Vita‐Salute San Raffaele University Milan Italy; ^18^ Gastroenterology and Gastrointestinal Endoscopy Unit IRCCS San Raffaele Scientific Institute Milan Italy; ^19^ Pancreatic Surgery Unit University of Verona Hospital Trust Verona Italy; ^20^ Radiology Department IRCCS San Raffaele Scientific Institute Milan Italy; ^21^ Pancreatic Surgery Unit, Department of Engineering for Innovation Medicine (DIMI) University of Verona Verona Italy; ^22^ Department of Biomedical Sciences Humanitas University Milan Italy; ^23^ Pancreatic Surgery Unit IRCCS Humanitas Research Hospital Milan Italy; ^24^ Pancreatic Surgery Unit Pancreas Translational and Clinical Research Center IRCCS San Raffaele Scientific Institute Milan Italy

**Keywords:** familial pancreatic cancer, genetic predisposition, NGS, pathogenic variants, surveillance

## Abstract

**Background:**

Little is known about the genetic background of individuals with familial pancreatic cancer (PC). Integrating germline testing into surveillance may uncover previously unrecognized hereditary susceptibility and expand prevention strategies beyond *BRCA* testing alone. This study evaluated the genetic landscape of high‐risk individuals due to familiality (HRI‐FHs) enrolled in a national surveillance program.

**Methods:**

Five hundred HRI‐FHs from seven centers underwent surveillance and germline testing with a 41‐gene NGS panel. Pathogenic/likely pathogenic variants (PGVs) and variants of unknown significance (VUS) were identified and correlated with clinical and imaging findings.

**Results:**

Overall, forty‐four (8.8%) out of 500 HRI‐FHs carried at least one PGV, including 3.4% in high‐penetrance genes (*ATM, BRCA1/2, PALB2, BRIP1*). Notably, 8 out of 17 (47%) of *ATM*, *BRCA1/2, PALB2* carriers would not have met the national testing criteria based solely on their family history. An additional 5.4% (27/500) carried PGVs in genes linked to other hereditary conditions (*CFTR, MUTYH, CTRC, SPINK1, APC*), and 39.6% harbored at least one VUS. PGV status, age, and female gender were independent predictors of radiological abnormalities. Two PCs were diagnosed, both in mutation‐negative individuals.

**Discussion:**

Integrating germline testing into surveillance redefines the management of familial PC. It uncovers hereditary susceptibility beyond classical criteria and supports cascade testing. PC also arises in mutation‐negative HRI. #NCT05724992.

## Key Summary

- Summarise the established knowledge on this subject
  ◦ The prevalence of high‐penetrance pathogenic germline variants in individuals with familial pancreatic cancer under surveillance is low, yet informative beyond traditional testing criteria.
- What are the significant and/or new findings of this study?
  ◦ Pancreatic cancer can also occur in high‐risk individuals without pathogenic germline variants, supporting surveillance regardless of genetic status.

**Abbreviations**
AISP: Associazione Italiana Studio Pancreas
HRI: High‐Risk Individual
HRI‐FH: High‐Risk Individual with a Family History of Pancreatic Cancer
IRFARPC: Italian Registry of Families At Risk of Pancreatic Cancer
ITPN: Intraductal Tubulopapillary Neoplasm
PC: Pancreatic Cancer
PGV: Pathogenic/likely pathogenic germline variant
PRECEDE: Pancreatic Cancer Early Detection Consortium
PROPH‐ITA: Familial Pancreatic Cancer PROPHylation Program in Italy
VUS: Variant of unknown significance

## 1 Introduction

Pancreatic cancer (PC) is projected to become the second leading cause of cancer‐related death by 2030 [1]. Its prognosis remains poor owing to several factors, including late diagnosis. In an effort to anticipate diagnoses, many initiatives have focused on identifying high‐risk individuals (HRIs) for active surveillance, such as those with multiple family members affected by PC (HRI‐HRs). The greater the number of affected relatives, the higher the lifetime risk of developing PC [2].

In approximately 10% of PC cases, pathogenic/likely pathogenic germline variants (PGVs) are hereditary cancer predisposing genes, most commonly *BRCA1, BRCA2, PALB2*, *CDKN2A*, and *ATM* [3, 4, 5, 6], and some hereditary pancreatitis genes (*PRSS1, SPINK1*) [7].

The reported prevalence of PGVs in cancer‐predisposing genes among HRI‐FHs undergoing PC surveillance ranges from 10% to 19% [3, 8, 9, 10], with peaks of 37.6% in selected cohorts of Jewish families [11].

Parallel to this, active surveillance programs for HRI‐FHs, such as those endorsed by the CAPS and PRECEDE consortia and other national registries [12, 13], offer an unprecedented opportunity to integrate systematic germline testing. Indeed, testing HRI‐FHs who are already under surveillance may not only identify carriers of well‐established high‐penetrance genes but also unveil variants conferring susceptibility to other hereditary cancer syndromes or to multiorgan predisposition. Additionally, in some surveillance contexts, PC seems to arise almost only in mutation carriers [14, 15], differentiating the risk profile of HRIs surveilled.

In 2015, a national registry for HRI was launched in Italy under the auspices of the Italian Association for the Study of the Pancreas (AISP). Results of the registry have already been published, revealing clinically relevant findings related to PCs, including eight cases detected under surveillance (three of which were Stage I and five of which were resected) as well as numerous other cystic and solid lesions [16, 17, 18].

The registry does not include standardized genetic testing for HRI‐FH cases. To address this limitation, a prospective parallel study was launched, the “Familial Pancreatic Cancer PROPHilation Program In Italy” (PROPH‐ITA). This initiative also aims to overcome the limitations of current multi‐society national guidelines, which recommend genetic testing only for HRI‐FHs with either two first‐degree relatives with PC or three relatives with PC up to the third‐degree kinship, and limited to a minimal panel targeting the *BRCA1* and *BRCA2* genes [19]. This study aims to investigate the genetic background of HRI‐FH cases by conducting buccal swab tests for assessing germline predisposition.

This manuscript presents an interim analysis of the first 500 HRI‐FHs tested, with a focus on the prevalence of PGVs, variants of unknown significance (VUS), and an analysis of imaging findings, by comparing carriers and non‐carriers.

## 2 Methods

### 2.1 Study Design and Case Recruitment

The study adheres to the guidelines for Strengthening the Reporting of Observational Studies in Epidemiology (STROBE) [20], was conducted in accordance with the principles of the Declaration of Helsinki, and was approved by the Institutional Review Board (#4040CESC) of the coordinating center and the Ethics Committee at each participating center. Written informed consent was obtained from all participants prior to inclusion in the study. The structure of the Italian Registry of Families At Risk of Pancreatic Cancer (IRFARPC) has already been published [16, 21]. The registry includes HRI‐FHs and HRI‐PGV carriers with and without a family history of PC. For this study, only HRI‐FHs undergoing active surveillance were prospectively enrolled and underwent genetic testing using a buccal swab, as described elsewhere [16], after providing consent. HRI‐FH was defined as having two relatives with PC in the same lineage, one of whom is a first‐degree relative, or having three cases in the same lineage, regardless of the degree (up to the third). As detailed elsewhere, the age limit to start surveillance in HRI‐FHs is 45 years or 10 years less than the youngest affected family member [16, 21]. When the enrollment and age criteria are met, subjects undergo annual surveillance using either magnetic resonance cholangiopancreatography or endoscopic ultrasound according to patient and center preference.

The study commenced in December 2022, and as of the database inception in February 2025, seven IRFARPC‐affiliated centers (listed in Supporting Information S1) of the 33 centers joining IRFARPC were actively recruiting participants in PROPH‐ITA. Each center received approval from the local Institutional Review Board. The study was registered at *Clinicaltrials.gov* (#NCT05724992) and funded solely by patient associations, benefactors, or dedicated fundraising events.

### 2.2 Genetic Analysis and Consultation

Gene selection was made after a literature review of the terms “familial pancreatic cancer,” “pancreatic cancer genetic predisposition,” and “pancreatic cancer mutated genes.” A 41‐gene panel was selected based on a review of the literature. It included: *APC, ASXL1, ATM, BAP1, BARD1, BMPR1A, BRCA1, BRCA2, BRIP1, CASR, CDK4, CDKN2A, CFTR, CHEK2, CPA1, CTRC, DNMT3A, EPCAM, FANCC, MEN1, MLH1, MSH2, MSH4, MSH6, MUTYH, NBN, NF1, PALB2, PALLD, PMS2, POLN, POLQ, PRSS1, PTEN, RAD51 C, RAD51D, SMAD4, SPINK1, STK11, TET2*, and *TP53*. These genes were associated with low‐, intermediate‐, or high‐penetrance. Some of them have been recently reported in PC patients with unclear significance (e.g., *ASXL1*, *TET2*, and *POLN*) [4]. In addition, the panel covered hereditary chronic pancreatitis genes (*CASR, CFTR, CPA1, CTRC, PRSS1,* and *SPINK1*) and other exploratory analyses on less investigated genes (e.g., *MSH4*).

A buccal swab was chosen to enhance participant compliance by providing sufficient DNA for NGS while avoiding the logistical challenges and perishability associated with blood samples. The swabs were collected and shipped to an accredited laboratory (Genomica s.r.l., Rome, Italy) where all the analyses were blinded to any proband's data. Genomic DNA was extracted from the biological samples, followed by whole‐exome sequencing using NGS technology on a Thermo Fisher platform (Thermo Fisher Scientific, Germering, Germany). The target coverage of the test exceeded 150x.

The analyzed variants across the 41 genes included single‐nucleotide variants (SNVs) and short insertions and deletions (indels) up to 20 base pairs in length, located within ± 5 nucleotides of exon boundaries or known splicing sites. The test was unable to detect mutations in intronic regions beyond this range, dynamic mutations such as triplet repeat expansions, larger structural variants exceeding 20 base pairs, or germline mosaicisms. Only variants classified as pathogenic (class 5), likely pathogenic (class 4), or variants of uncertain clinical significance (VUS, class 3) according to the ACMG guidelines were reported, with classes 4 and 5 further reported as PGVs. These classifications were based on guidelines, scientific literature and data from major databases such as the Human Gene Mutation Database, ClinVar, Varsome, OMIM, GeneReview, dbSNP, and NCBI. The obtained gene sequences were analyzed using bioinformatics analysis.

The interpretation of mutations was based on the most current knowledge available at the time of analysis and report generation. When existing scientific research, including published studies and experimental data, did not provide sufficient evidence to determine the impact of the mutation on protein function or disease risk, in silico prediction tools were utilized. Results were communicated by email or telephone by the center's principal investigator for negative results. Conversely, they were discussed during genetic counseling if they tested positive for PGVs or VUS.

Genetic counseling was provided to discuss the clinical implications of the results, explore potential management strategies, and propose cascade testing. Additionally, patients received a written report and were informed that the interpretation of VUS may change over time due to new scientific discoveries. In cases of VUS, individuals were referred to local genetic counselors to monitor for future reclassification updates. For families with a living relative affected by PC, a segregation study of the PGV was offered to confirm or exclude the correlation between mutation and family history. In cases of PGVs, cascade testing was prioritized for first‐degree relatives. Second‐degree relatives were included as appropriate, depending on family structure, variant penetrance, and initial testing outcomes.

Since traditional genetic counseling models are time‐intensive and resources are sparse, a mainstream model was developed, so comprehensive genetic counseling was offered only if results revealed pathogenic, likely pathogenic, or VUS.

### 2.3 Data Collection and Definitions

Data collected included demographic information, personal and family history of malignancies, results of surveillance imaging procedures, and genetic test results. The personal and family history of malignancies was self‐reported by the subjects during direct interviews conducted by the investigators, with no proxies used, and for first‐degree relatives, medical records were deemed mandatory. Genetic data were anonymized before being sent to the laboratory.

Radiological abnormalities were classified as cystic (any cyst > 5 mm, presumably diagnosed as mucinous cystic neoplasm or cystic neuroendocrine tumor) or solid. Given that most HRI‐FHs had only one follow‐up visit, radiological abnormalities were investigated and compared across groups using only baseline imaging.

All data were transferred into a dedicated electronic case record form and exported into an Excel sheet.

### 2.4 Statistical Analysis

Continuous variables were expressed as medians with interquartile range (IQR) and categorical variables were presented as frequencies with percentages.

A multivariable logistic regression analysis was used to identify predictors of germline mutations using personal and family history of malignancies, including the age of cancer diagnosis.

The association between the presence of radiological abnormalities at baseline screening and genetic status was investigated using a binary logistic regression model. A multivariable model was constructed using clinically relevant variables. The *p*‐values are presented with odds ratios (ORs) and 95% confidence intervals (CI), as appropriate. Statistical significance was determined as a *p*‐value < 0.05. The overall proportion of missing data was low and did not affect the primary outcomes. No imputation methods were applied, as the extent of missingness was minimal and unlikely to introduce bias. All statistical analyses were performed using SPSS 29 (IBM Corp., Armonk, NY, USA).

## 3 Results

**FIGURE 1:**
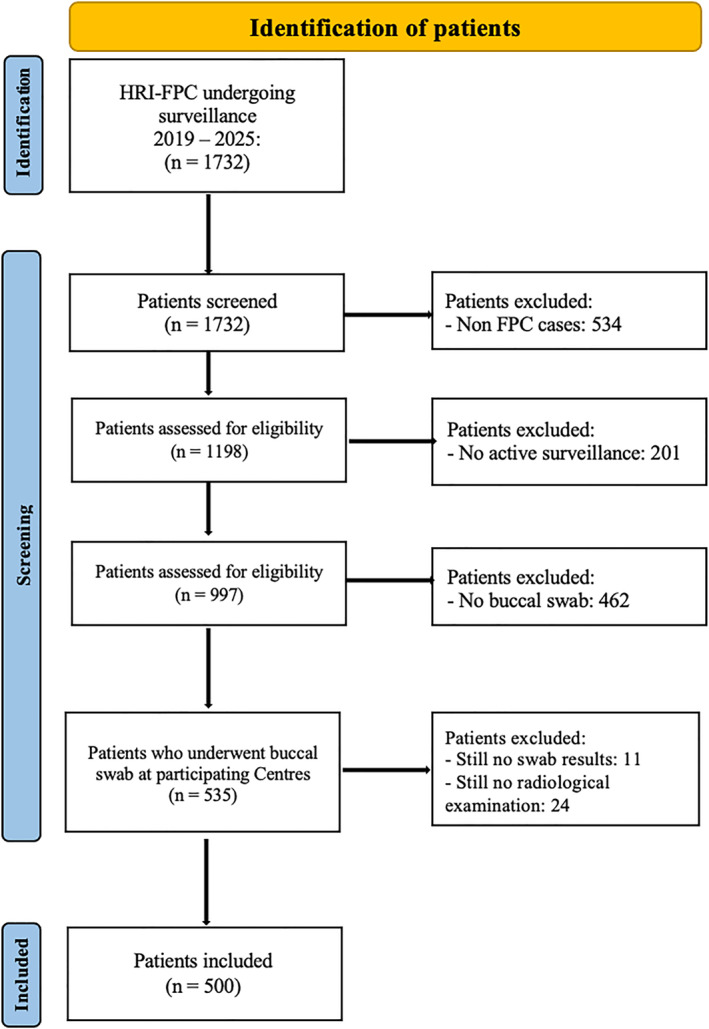
Study flowchart.

At the time of the study analysis, the IRFARPC included 1732 HRIs. Among them, 1198 were HRI‐FHs, and 997 had already undergone active surveillance. Five hundred HRI‐FHs (41.7% of all HRI‐FH cases) were tested using a buccal swab at the seven participating Centers. The study flowchart is shown in Figure 1.

The median age was 54 (IQR 49–60) with a female prevalence of 60.6%. The median number of relatives with PC was 2 (IQR 2–3); 79.2% of HRI‐FHs had at least one first‐degree and one second‐degree relative with PC, and 27.4% had at least two first‐degree relatives with PC. Table 1 presents the general features of the study population.

**TABLE 1:** Study population (*n* = 500).

Variable	*n* = 500
Age, years (median, IQR)	54 (49–60)
Sex, *n* (%)	
Female	303 (60.6)
Male	197 (39.4)
BMI (median, IQR)	23.9 (21.8–27.1)
Smoking habit (current or former), *n* (%)	156 (31.5)
Alcohol habit (current or former), *n* (%)	166 (33.2)
Diabetes mellitus, *n* (%)	14 (2.8)
Family history of PC (same lineage), *n* (%)^a^	
2 first‐degree relatives	137 (27.4)
1 first‐degree and one second‐degree	396 (79.2)
≥ 3 relatives	124 (24.8)
Number of relatives with PC (median, IQR)	2 (2–3)
Most frequent age range of the YRA^b^	60–69
Personal history of any cancer, *n* (%)	43 (8.6)

^a^ Values may exceed 100% because participants could meet multiple family history criteria.

^b^ Youngest affected relative.

Abbreviation: PC, pancreatic cancer.

All individuals to whom testing was proposed consented to provide a buccal swab and to receive their genetic results.

### 3.1 Study Findings: PGV

Among the 500 HRI‐FHs undergoing surveillance, 44 (8.8%) carried at least one PGV. Of these, one individual harbored two simultaneous PGVs, located in *ATM* and *PALB2* genes, respectively. All mutations were heterozygous. PGVs in high‐penetrance PC susceptibility genes were identified in 17 HRI‐FHs (3.4%). They included mutations in *ATM* (*n* = 8, 1.6%), *BRCA2* (*n* = 6, 1.2%), *BRCA1* (*n* = 2, 0.4%), and one each in BRIP1 (0.2%) and *PALB2* (the *PALB2* mutation co‐occurred with an *ATM* one). The remaining 27 HRI‐FHs harboring PGVs (5.4% of the cohort) carried mutations in genes associated with other conditions. Specifically, 25 HRI‐FHs were carriers for variants in genes linked to autosomal recessive syndromes (*CFTR*, *n* = 17, 3.4%) and *MUTYH* (*n* = 8, 1.6%). Additionally, two individuals carried mutations associated with an increased risk of hereditary pancreatitis (in the *CTRC* and *SPINK1* genes, one case each, 0.2%), and one individual carried a mutation in the *APC* gene.

In fourteen HRI‐FHs (2.8%), a PGV co‐occurred with a VUS. Two carriers (4.4%) had a prior history of malignancy (one breast cancer and one non‐Hodgkin lymphoma). Table 2 provides a detailed description of PGV carriers.

**TABLE 2:** Pathogenic germline variants, all in heterozygosity and class 4 or 5 according to the ACMG guidelines [22].

Case ID	Age, y	Gender	Gene	Nucleotide Change	Amino Acid Change	Function (ACMG classification)	Significance	FH of PC	pH of Cancer, Type	Fulfills National Genetic Testing Guidelines^c^
High‐penetrance PC susceptibility genes										
377^a^	55	M	*ATM*	c.2720_2723del	p.Leu906_Cys907insTer	Nonsense (5)	Cancer predisposition syndrome	1 FDR, 3 SDR	No	Yes
1071	61	M	*ATM*	c.3346 C > T	p.Gln1116Ter	Intron variant (4)	Cancer predisposition syndrome	1 FDR, 1 SDR	No	No
1076	49	M	*ATM*	c.2250 G > A	p.Lys750 =	Synonymous variant (4)	Cancer predisposition syndrome	1 FDR, 1 SDR	Gastric GIST	No
1198	49	M	*ATM*	c.748 C > T	p.Arg250Ter	Nonsense (5)	Cancer predisposition syndrome	1 FDR, 1 SDR	No	No
1201^a^	75	M	*ATM*	c.748 C > T	p.Arg250Ter	Nonsense	Cancer predisposition syndrome	2 FDR	No	Yes
1200	53	F	*ATM*	c.748 C > T	p.Arg250Ter	Nonsense	Cancer predisposition syndrome	2 FDR	No	Yes
1274	73	M	*ATM*	c.7517_7520del	p.Arg2506ThrfsTer3	Intron variant (5)	Cancer predisposition syndrome	1 FDR, 1 SDR	No	No
232^b^	40	F	*ATM*	c.1212_1213del	p.Gln404_Asn405insTer	Frameshift variant (5)	Cancer predisposition syndrome	1 FDR, 1 SDR	No	No
153	49	F	*BRCA1*	c.1088del	p.Asn363 fs	Frameshift variant (5)	Cancer predisposition syndrome	2 FDR	No	Yes
154	50	F	*BRCA1*	c.1088del	p.Asn363 fs	Frameshift variant	Cancer predisposition syndrome	2 FDR	No	Yes
208	63	F	*BRCA2*	c.7180 A > T	p.Arg2394Ter	Nonsense (5)	Cancer predisposition syndrome	1 FDR, 1 SDR, 1 TDR	No	Yes
437	47	M	*BRCA2*	c.8878 C > T	p.Gln2960Ter	Nonsense (5)	Cancer predisposition syndrome	1 FDR, 1 SDR	No	No
438	43	M	*BRCA2*	c.8878 C > T	p.Gln2960Ter	Nonsense	Cancer predisposition syndrome	1 FDR, 1 SDR	No	No
1303^c^	62	M	*BRCA2*	c.4222 C > T	p.Gln1408Ter	Nonsense (5)	Cancer predisposition syndrome	1 FDR, 1 SDR	No	No
1518	54	M	*BRCA2*	c.5682 C > G	p.Tyr1894Ter	Nonsense (5)	Cancer predisposition syndrome	1 FDR, 3 SDR, 1 TDR	No	Yes
1667^d^	72	F	*BRCA2*	c.2830 A > T	p.Lys944Ter	Nonsense (5)	Cancer predisposition syndrome	3 FDR, 1 SDR	Breast	Yes
1201^a^	72	M	*PALB2*	c.1317del	p.Phe440 fs	Frameshift variant (5)	Cancer predisposition syndrome	2 FDR	No	Yes
1751^e^	54	F	*BRIP1*	c.1343 G > A	p.Trp448Ter	Nonsense (5)	Cancer predisposition syndrome	1 FDR, 1 SDR	No	No
Genes associated with autosomal recessive syndromes										
636^f^	51	F	*CFTR*	c.2051_2052delinsG	p.Lys684 fs	Frameshift variant (5)	Carrier of AR disorder	1 FDR, 1 SDR	No	No
157	53	F	*CFTR*	c.1585‐1G > A	—	Splice acceptor variant (5)	Carrier of AR disorder	1 FDR, 1 SDR	NHL	No
267^g^	39	F	*CFTR*	c.350 G > A	p.Arg117His	Missense (5)	Carrier of AR disorder	2 FDR	No	Yes
523	57	M	*CFTR*	c.1521_1523del	p.Phe508del	Loss of function variant (5)	Carrier of AR disorder	1 FDR, 1 SDR	No	Yes
737	49	M	*CFTR*	c.1521_1523del	p.Phe508del	Loss of function variant	Carrier of AR disorder	1 FDR, 1 SDR	No	No
1430	70	M	*CFTR*	c.1521_1523del	p.Phe508del	Loss of function variant	Carrier of AR disorder	2 FDR	No	Yes
1717	48	M	*CFTR*	c.1521_1523del	p.Phe508del	Loss of function variant	Carrier of AR disorder	1 FDR, 2 SDR	No	Yes
1696^h^	63	F	*CFTR*	c.489 + 3A > G	—	Intron variant (4)	Carrier of AR disorder	1 FDR, 1 SDR	No	No
670^i^	61	M	*CFTR*	c.3154 T > G	p.Phe1052Val	Missense (4)^b^	Carrier of AR disorder	1 FDR, 2 SDR	No	Yes
671^i^	59	F	*CFTR*	c.3154 T > G	p.Phe1052Val	Missense	Carrier of AR disorder	1 FDR, 2 SDR	No	Yes
672	55	F	*CFTR*	c.3154 T > G	p.Phe1052Val	Missense	Carrier of AR disorder	1 FDR, 2 SDR	No	Yes
1045^l^	67	M	*CFTR*	c.1013 C > T	p.Thr338Ile	Missense (4)	Carrier of AR disorder	2 FDR	No	Yes
1048	52	F	*CFTR*	c.3095 A > G	p.Tyr1032Cys	Missense (4)	Carrier of AR disorder	1 FDR, 1 SDR	No	No
1372	76	F	*CFTR*	c.3909 C > G	p.Asn1303Lys	Missense (4)	Carrier of AR disorder	2 FDR, 1 SDR	No	Yes
1327	75	F	*CFTR*	c.2991 G > C	p.Leu997Phe	Missense (4)	Carrier of AR disorder	2FDR, 1 SDR	No	Yes
1455	51	F	*CFTR*	c.2991 G > C	p.Leu997Phe	Missense	Carrier of AR disorder	1 FDR 3 SDR	No	Yes
1470	63	F	*CFTR*	c.2991 G > C	p.Leu997Phe	Missense	Carrier of AR disorder	1 FDR, 2 SDR	No	Yes
390	69	M	*MUTYH*	c.650 G > A	p.Arg217His	Missense (5)	Carrier of AR disorder	2 FDR	No	Yes
160	43	F	*MUTYH*	c.1103 G > A	p.Gly368Asp	Missense (4)	Carrier of AR disorder	4 SDR	No	Yes
908	67	M	*MUTYH*	c.1103 G > A	p.Gly368Asp	Missense	Carrier of AR disorder	1 FDR, 1 SDR	No	No
974^m^	60	F	*MUTYH*	c.1103 G > A	p.Gly368Asp	Missense	Carrier of AR disorder	2 FDR	No	Yes
1423	54	F	*MUTYH*	c.1103 G > A	p.Gly368Asp	Missense	Carrier of AR disorder	1 FDR, 1 SDR	No	No
666	66	F	*MUTYH*	c.1350GGA[1]	p.Glu452del	Inframe deletion (4)	Carrier of AR disorder	1 FDR, 1 SDR	No	No
668	67	M	*MUTYH*	c.1350GGA[1]	p.Glu452del	Inframe deletion	Carrier of AR disorder	1 FDR, 1 SDR	No	No
1606	66	F	*MUTYH*	c.452 A > G	p.Tyr151Cys	Missense (4)	Carrier of AR disorder	1 FDR, 2 SDR	No	Yes
Genes associated with hereditary pancreatitis risk										
204	58	F	*CTRC*	c.760 C > T	p.Arg254Trp	Missense (5)	CP risk factor	1 FDR, 1 SDR	No	No
1651^n^	54	F	*SPINK1*	c.101 A > G	p.Asn34Ser	Missense (5)	CP risk factor	1 FDR, 2 SDR	No	Yes
Genes associated with other conditions										
665	75	M	*APC*	c.3920 T > A	p.Ile1307Lys	Missense (5)	Colorectal cancer risk factor in AJ	2 FDR	No	Yes

*Note:* The following individuals also had a variant of unknown significance in the following genes: ^a^ *APC* (c.737 C > T; p.Ser246Phe); ^b^ *PALLD* (c.1297 G > A; p.Gly433Arg); ^c^ *POLQ* (c.6992 A > G; p.Tyr2331Cys); ^d^ *PALLD* (c.1965–12879C > G); ^e^ *ASXL1* (c.4281_4282delinsGC; p.Ser1428Pro); ^f^ *ATM* (c.4673 C > T; p.Thr1558Met); ^g^ *MLH1* (c.1693 A > T; p.Ile565Phe); ^h^ *CTRC* (c.−59C > T); ^i^ *POLQ* (c.7393 G > A; p.Glu2465Lys); ^l^ *POLN* (c.2308 + 6 A > G); ^m^ *CFTR* (c.91 C > T; p.Arg31Cys); ^n^ *CFTR* (c.249 C > G; p.Pe83Leu).

Abbreviations: AJ, Ashkenazi Jewish; AR, autosomal recessive; FDR, first‐degree relative; FH, family history; GIST: gastrointestinal stromal tumor; NHL, non‐Hodgkin lymphoma; PC, pancreatic cancer; SDR, second‐degree relative; TDR, third‐degree relative.

^a^ Same participant.

^b^ For this variant, ClinVar reports conflicting interpretations of pathogenicity (last evaluated March 2024), with submissions ranging from “uncertain significance” to “likely pathogenic,” depending on the clinical context.

^c^ Germline testing for BRCA1/BRCA2 is recommended in HRI individuals who have two first‐degree relatives or at least three relatives (up to the third degree) affected by PC in the same lineage.

In the multivariable logistic regression analysis, neither personal nor family history data predicted the presence of PGVs, even when analyzing the entire gene panel or restricting it to homologous recombination repair (HRR) genes (data not shown).

Twenty‐three out of 44 HRI‐FHs (52.3%) with PGV fulfilled the national recommendations for genetic testing based on PC family history.

### 3.2 VUS Identified

A total of 261 VUS were identified in 198 individuals, for a total prevalence of VUS of 39.6% in HRI‐FHs (Figure S1, Supporting Information S1). The genes that were more commonly found harboring VUS were *CFTR* (*n* = 33, 12.6%), *POLQ* (*n* = 25, 9.5%), *APC* (*n* = 23, 8.8%), *MUTYH* (*n* = 11, 4.2%), *ATM* (*n* = 11, 4.2%), and *BRCA2* (*n* = 10, 3.8%). Supporting Information S2 lists the identified VUS.

### 3.3 Imaging Findings: Overall

200 participants (40%) who underwent baseline imaging only, 78 completed two rounds of imaging (15.6%), while the remaining 222 (44.4%) had at least three rounds of imaging.

Radiological abnormalities were found in 156 participants (31.2%). They were cystic in 149 (29.8%) cases, solid in six (1.2%), and solid and cystic (co‐occurring) in one (0.2%).

Among the six HRI‐FHs diagnosed with solid lesions, four were identified on baseline imaging. All were pancreatic neuroendocrine tumors < 2 cm and were kept under surveillance. Among them, three patients tested negative for mutations, while one had a VUS in *APC* (c.6627 T > A, p.Ile2209Met). The remaining two cases (0.4% of the total) were two incident PCs, diagnosed at the second follow‐up at 13 and 14 months, after a baseline imaging deemed negative for significant pancreatic abnormalities.

The first case of PC was located in the pancreatic head. The patient, a 65‐year‐old female with two relatives affected by PC (mother and maternal aunt), was found to have a VUS in *CASR* (c.992 G > A, p.Arg331Gln). She initially received chemotherapy with FOLFIRINOX (Folinic Acid, Fluorouracil, Irinotecan, and Oxaliplatin) for neoadjuvant intent due to tumor dimensions (38 mm) and nodal involvement. The patient progressed to liver metastases 5 months after diagnosis and died 2 months later. The re‐evaluation of baseline imaging confirmed the absence of any pancreatic alteration.

The second case of PC was located in the tail of the pancreas. The patient, a 69‐year‐old female with two first‐degree relatives with PC (father and brother), underwent left pancreatectomy and splenectomy for a 1 cm solid nodule at the pancreatic tail, and final pathology revealed a pancreatic intraductal tubulopapillary neoplasm (ITPN) with an invasive component (Size of the whole lesion: 1.2 cm, size of the invasive component for T assessment: 4 mm; pT1aN0M0, Stage 1a). In this case, her swab was negative for mutations, and a re‐evaluation of the baseline imaging identified focal atrophy of the pancreatic tail.

### 3.4 Imaging Findings: Comparison Between Carriers and Non‐carriers

When considering the presence of any radiological abnormalities, whether incident or prevalent, no differences were observed between carriers and non‐carriers. This held true when analyzing all mutations (including VUS), only PGVs, and when restricting the analysis to HRR genes (Table S1).

### 3.5 Predictors of Radiological Anomalies at Baseline Screening

Clinical predictors of the presence of radiological findings at baseline screening are shown in Table 3. On bivariate logistic regression modeling, age (OR 1.084 [95% CI 1.055–1.113], *p* < 0.001), female sex (OR 1.778 [95% CI 1.142–2.769], *p* = 0.011), and PGV carrier (OR 1.585 [95% CI 1.021–2.460], *p* = 0.04) were independently associated with the presence of anomalies at baseline imaging.

**TABLE 3:** Predictors of radiological abnormalities at baseline screening.

Variable	Univariate analysis		Multivariate analysis	
	OR (95% CI)	*p* value	OR (95% CI)	*p* value
Age	1.074 (1.049–1.100)	**< 0.001**	1.084 (1.055–1.113)	**< 0.001**
Female	1.474 (0.991–2.194)	0.056	1.778 (1.142–2.769)	**0.011**
Smoking habit (current or former)	1.048 (0.696–1.578)	0.822	0.930 (0.588–1.474)	0.759
≥ 2 first‐degree relatives	1.368 (0.892–2.097)	0.151	0.846 (0.516–1.387)	0.507
Non‐carrier	1 (ref)	—	—	—
Carrier of PGV	2.026 (0.928–4.442)	0.076	2.369 (1.022–5.491)	**0.044**
Carrier of VUS	1.284 (0.862–1.914)	0.219	1.344 (0.873–2.069)	0.179
Carrier of PGV in HRR genes	1.133 (0.380–3.375)	0.822	1.393 (0.432–4.488)	0.579

*Note:* Values in bold are statistically significant.

Abbreviations: HRR, homologous recombination repair; PGV, pathogenic germline variants; VUS, variant of unknown significance.

## 4 Discussion

This multicenter study presents one of the largest prospective genetic characterizations of HRI‐FH individuals undergoing active surveillance within a national program. Testing was performed using a broad 41‐gene panel encompassing high‐, moderate‐, and low‐penetrance genes, thus offering a comprehensive snapshot of the hereditary landscape associated with familial pancreatic cancer. Most genes included have been previously studied in familial PC or PC predisposition [4, 6, 9, 23], with some additional genes added for exploratory purposes.

Although only 8.8% of participants carried a PGV, only 3.4% harbored PGVs in high‐penetrance genes. This apparent “low yield” was lower than that observed in other cohorts of HRI‐FHs [4, 5, 6, 8, 10, 24] and similar to that reported in other studies [9, 10, 11, 25]. It must be considered that, in our patient population, where the index PC case was seldom tested, Mendelian segregation may have caused a 50% signal loss. Additionally, we extended our testing to cases with a weaker family history than the classical definition, and some authors reported differences in PGVs when the familiarity signal was diluted [25]. Finally, we did not perform large genomic rearrangements in the *BRCA* genes. However, the latter would have primarily affected *BRCA1,* which is typically less frequently mutated in this context.

However, our findings still underscore the importance of integrating germline testing within PC surveillance, as an opportunity to identify previously unrecognized hereditary cancer predispositions, often beyond the cancer itself. Indeed, 38% of *BRCA1/2* carriers in this cohort did not meet national testing criteria based solely on a “classical” family history (e.g., HRI‐FHs with at least two first‐degree relatives with PC), confirming either the poor sensitivity of traditional pedigree‐based models for selecting candidates for genetic testing, as reported in other oncological contexts [26, 27]. Overall, these results align with the PRECEDE initiative, which emphasizes that germline testing in HRI‐FHs broadens the detection of actionable mutations and facilitates cascade testing and organ‐specific prevention for relatives [28].

The PGVs found in other genes, including those associated with recessive syndromes (*CFTR, MUTYH*), hereditary pancreatitis (*CTRC, SPINK1*) or other low‐risk conditions (the APC the p.Ile1307Lys variant, which confers moderate colorectal cancer risk in Ashkenazi Jewish individuals [29, 30]), may be considered as secondary bycatch of the explorative nature of the study that in the future might become valuable foundation for studies investigating gene–environment and gene–gene interactions in pancreatic carcinogenesis. Equally relevant is the remarkably high prevalence of VUS (39.6%) across multiple genes. As new data emerge, many VUS will be reclassified as (likely) pathogenic [31, 32], progressively transforming today's uncertainty into tomorrow's knowledge.

From a clinical perspective, the most relevant findings were the diagnosis of two PCs in patients who tested negative for PGV and the absence of differences in imaging abnormalities between carriers and non‐carriers. This supports the current NCCN recommendations to offer surveillance to all HRI‐FH individuals, regardless of their mutation status and remains a central topic of debate. Indeed, while long‐standing Dutch [14] and German [15] registries have reported that PC develops only or almost only [33], in mutation carriers, several other studies have also documented PC in non‐genetically predisposed individuals [17, 33, 34, 35, 36, 37]. Comparison among registries should be done with caution, given that variability exists in practice patterns in terms of surveillance criteria and imaging modalities and HRI risk profile [38]. While longer follow‐up is required to define long‐term risk trajectories, our observation suggests that mutation status should not be used to de‐escalate or withhold surveillance. Surveillance strategies should therefore continue to include both mutation carriers and mutation‐negative HRI‐FH, and a negative germline test should not be interpreted as a marker of clinical reassurance. Familial aggregation of PC likely reflects a combination of undiscovered genetic factors, polygenic risk, and shared environmental or lifestyle exposures. In this context, surveillance remains the only strategy capable of mitigating risk, regardless of the results of current germline testing.

Some limitations should be acknowledged. First, family‐level data and the distribution of variants within families were not available. Second, CNV and large rearrangement analyses were not performed. Third, segregation analyses were limited. Fourth, from a clinical standpoint, the lack of a standardized imaging protocol across the participating centers might have masked imaging differences between carriers and non‐carriers.

In summary, we demonstrate that surveillance programs for PC are no longer limited to being diagnostic tools aimed solely at early detection; they have evolved into strategic platforms to identify mutations across various cancer types, facilitate family counseling and cascade testing, and enhance the molecular understanding of familial cancer syndromes. Our data further support the continuation of PC surveillance in individuals with familial PC, irrespective of germline mutation status. Until more refined tools for risk stratification become available, excluding individuals from surveillance based solely on a negative genetic test appears unwarranted.

## Funding

This study was supported by Fondazione Nadia Valsecchi Onlus, Associazione Oltre La Ricerca.

Gli Amici di Mario Sala, iSempreVivi Onlus, and #IOVADOALMASSIMOBIKE, led by Massimo Canonica, MD, who supported the project until his passing due to pancreatic cancer. The funding sources played no role in the study design, data collection and analysis, decision to publish, or preparation of the manuscript.

The study results have been presented at the following congresses: Italian Association for the Study of the Pancreas annual meetings, Bergamo, Italy, September 2024 and September 2025; European Pancreatic Club, Santiago de Compostela, Spain, June 2024; UEG Week, Wien, Austria, October 2024; PRECEDE Consortium annual meeting, San Diego, USA, November 2025.

## Conflicts of Interest

SP received consultancy honoraria from AlphaTau Medical and ClearNoteHealth, and speaker fees from Fresenius Kabi. ES received consultancy honoraria from AlphaTau Medical. SC is a consultant for Olympus and Boston Scientific. GM received consultancy honoraria from OncoSil. GC received consultancy honoraria from Amgen, Boston Scientific, Dr. Falk, Pangenix, and Viatris. LA received consultancy honoraria from Boston Scientific, Pentax, and Viatris. CC has been a lecturer for Steris and a consultant for Mediglobe. CB has been a lecturer for Boston Scientific, Steris, Fujifilm, and is a consultant for Canon. DB is employed by the private laboratory (Genomica s.r.l.), which is involved in DNA extraction and NGS analysis as part of this study. The oher authors have nothing to declare.

## Supporting information


Supporting Information S1

Supporting Information S2

## Data Availability

The data that support the findings of this study are available from the corresponding author upon reasonable request.
