# Correction to “Discovering Hereditary Risk Through Surveillance: A Prospective Genetic Analysis of Individuals With Familial Pancreatic Cancer”

**DOI:** 10.1002/ueg2.70288

**Published:** 2026-08-28

S. Paiella, E. Secchettin, L. Archibugi, et al., “Discovering Hereditary Risk Through Surveillance: A Prospective Genetic Analysis of Individuals With Familial Pancreatic Cancer,” *United European Gastroenterology Journal* 14, no. 1 (2026): e70187, https://doi.org/10.1002/ueg2.70187.

Maria Terrin was incorrectly attributed to the affiliation “Department of Biomedical Sciences, Humanitas University, Milan, Italy.” She was not affiliated with this university at the time of publication.

We apologize for this error.

