# Supplementary material for: Discovering Hereditary Risk Through Surveillance: A Prospective Genetic Analysis of Individuals With Familial Pancreatic Cancer
Source: United European Gastroenterol J. 2026 Feb 15;14(1):e70187. doi: 10.1002/ueg2.70187 (PMC12906649; doi:10.1002/ueg2.70187)
Supplement: Supplementary file 1 — Supporting Information S1 [file UEG2-14-e70187-s002.docx]

**Supplementary material 1**

*List of participating Centers*

- The General and Pancreatic Surgery Unit, Pancreas Institute, University of Verona, Verona:
- Pancreato-Biliary Endoscopy and Endosonography Division, Pancreas Translational and Clinical Research Center, and the Gastroenterology and Gastrointestinal Endoscopy Unit, IRCCS San Raffaele Scientific Institute, Vita-Salute San Raffaele University, Milan;
- Gastroenterology Department, Humanitas Research Hospital-IRCCS, Rozzano, Milan;
- Department of Surgical Oncology, IRCCS Istituto Tumori "Giovanni Paolo II", Bari;
- Department of Surgery, Oncology and Gastroenterology, University of Padua, Padua;
- Gastroenterology and Digestive Endoscopy Unit, Azienda USL - IRCCS di Reggio Emilia**,** Reggio Emilia;
- Gastroenterology and Digestive Endoscopy Unit, Forlì-Cesena Hospitals, AUSL Romagna, Forlì-Cesena.

**Table S1.** Radiological findings at baseline screening in carriers vs non-carriers.

|  | **Total** | **Normal** | **Uni- or multifocal cyst w/o WF/HRS** | **Cyst with WF** | **Solid lesion** |
| --- | --- | --- | --- | --- | --- |
| **Non-carrier, n (%)** | 256 (51.2) | 187 (73.0) | 66 (25.8) | 0 (0) | 3 (1.2) |
| **Carrier, n (%)** | 244 (48.8) | 159 (65.2) | 83 (34.0) | 1 (0.4) | 1 (0.4) |
| VUS | 198 (39.6) | 128 (64.6) | 68 (34.3) | 1 (0.5) | 1 (0.5) |
| PGV (any gene) | 44 (8.8) | 29 (65.9) | 15 (34.1) | 0 (0) | 0 (0) |
| PGV (any HRR genes) | 17 (3.4) | 12 (70.6) | 5 (29.4) | 0 (0) | 0 (0) |

WF: worrisome feature; HRS: high-risk stigmata; PGV: pathogenic germline variant; VUS: variant of unknown significance; Homologous Recombination Repair.

p-value adjusted for multiple comparisons (Bonferroni method)

p-value= 0.086 (carriers vs non-carriers)

p-value= 0.529 (non-carriers vs PGV vs VUS vs HRR genes)

p-value= 0.990 (PGV vs VUS vs HRR genes)

**Figure S1**. Variant of unknown significance (VUS) detected by buccal swabbing.

**
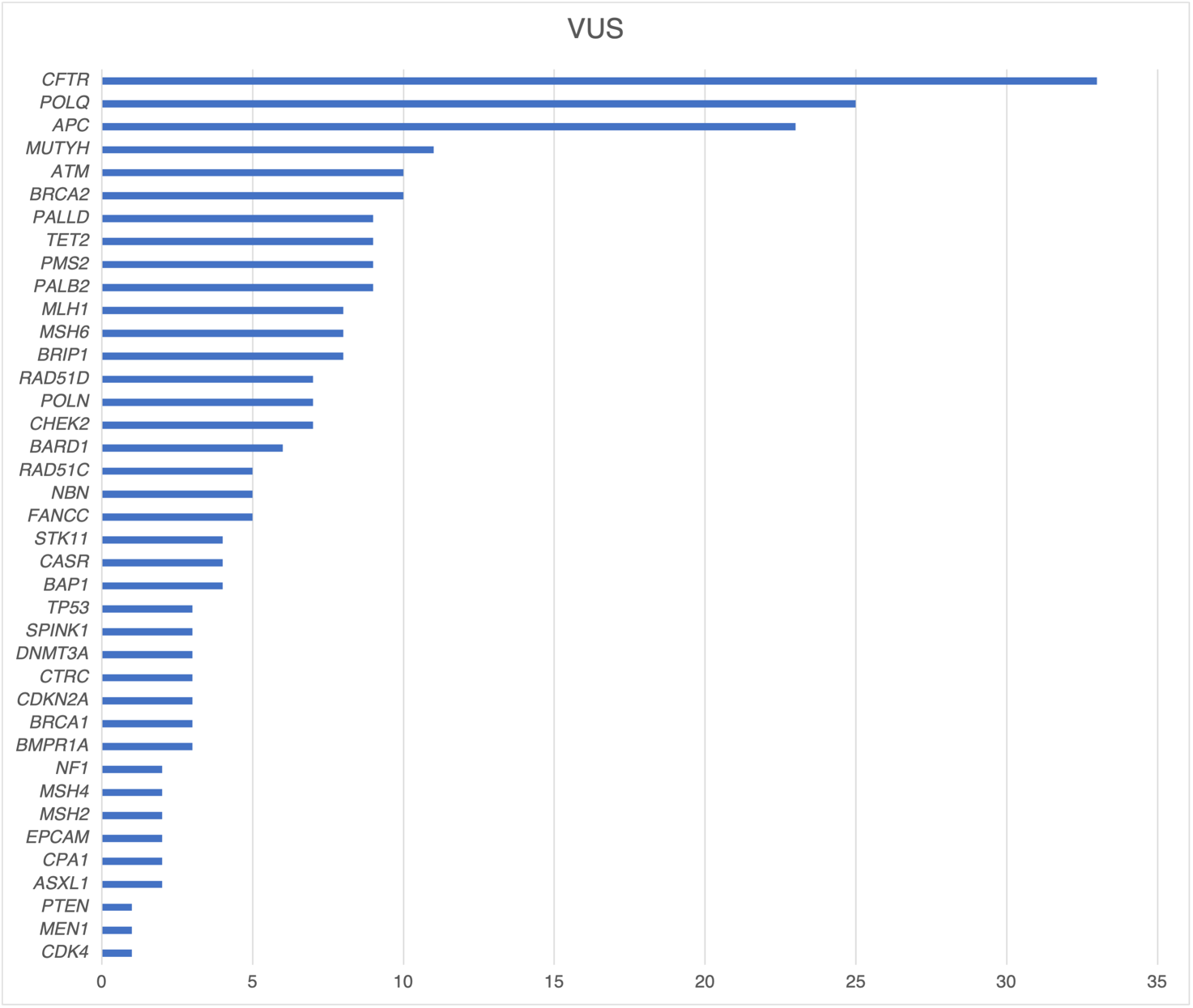
**
